# Prevalence of married women’s decision-making autonomy on contraceptive use and its associated factors in high fertility regions of Ethiopia: a multilevel analysis using EDHS 2016 data

**DOI:** 10.1186/s12889-023-15009-y

**Published:** 2023-01-11

**Authors:** Tadele Biresaw Belachew, Wubshet Debebe Negash, Desalegn Anmut Bitew, Desale Bihonegn Asmamaw

**Affiliations:** 1 Department of Health Systems and Policy, Institute of Public Health, College of Medicine and Health Sciences, University of Gondar, P.O.Box: 196, Gondar, Ethiopia; 2 Department of Reproductive Health, Institute of Public Health, College of Medicine and Health Sciences, University of Gondar, Gondar, Ethiopia

**Keywords:** Decision making, Contraceptive, Multilevel, Ethiopia

## Abstract

**Background:**

Women's independence in reproductive health is crucial to the health of mothers and children. Couples are, however, restricted from discussing their relationship openly. Regarding this, information about women’s decision-making autonomy is low in developing countries including Ethiopia. Therefore, this study was aimed to assess married women’s decision-making autonomy on contraceptive use in high fertility regions of Ethiopia.

**Methods:**

The study was based on secondary data analysis of the Ethiopian Demographic and Health Survey 2016 data. A total weighted sample of 1157 reproductive age women was included. A multilevel mixed-effect binary logistic regression model was fitted to identify the significant associated factors of decision making autonomy on contraceptive use. Statistical significance was determined using Adjusted Odds Ratio (AOR) with 95% confidence interval.

**Results:**

Overall prevalence of decision making autonomy on contraceptive use was observed to be 17.2% (15.1, 19.5). Women's age 25–34 (AOR = 3.19; 95% CI:1.55, 6.54), and 35–49 (AOR = 3.59; 95% CI: 1.5, 8.36), secondary and above educational level (AOR = 3.38; 95% CI: 1.07, 10.67), being married before 18 years (AOR = 0.42; 95% CI:0.26, 0.68), being Muslim in religion (AOR = 0.47; 95% CI: 0.23, 0.98), women being in urban area (AOR = 2.73; 95% CI: 1.97, 6.35), and community media exposure (AOR = 1.85; 95% CI: 1.15, 2.48) were associated with decision making autonomy on contraceptive use.

**Conclusion:**

Women’s decision-making autonomy on contraceptive use in this study was low. Age of mothers, educational status of mothers, age at first marriage, residence, religion, and community media exposure were significant factors. Therefore, the government should promote women’s autonomy on contraceptive use as an essential component of reproductive health rights through mass media, educating, with particular attention for, youth women, and women living in rural settings.

## Background

Decision-making autonomous is the ability of women to make their own decisions on matters that concern them [1]. To achieve better maternal and child health outcomes, women need to make their own reproductive health decisions [2, 3]. Nevertheless, women are unable to access reproductive health services, including contraceptives, because of gender-based power inequalities [4]. As a result of women's autonomy when it comes to making health decisions, they are more likely to get access to health information and use reproductive health services [5]. In the former studies, higher levels of women's decision-making autonomy were associated with increased contraceptive use [2, 6–8]. A women's autonomy in making decisions is also related to sexual reproductive health (SRH) issues [6, 9–11].

There have been studies in different parts of Africa showing that women are less autonomous when it comes to making decisions about contraceptive and other sexual health issues, because of sociocultural factor affecting their access and gender norms [6, 7, 11–17]. Globally, only 55% of married women have decision-making autonomy on SRH issues, with 36% in Sub-Saharan Africa [18] and 21.6% women's autonomy over contraceptive use in Ethiopia [19].

In previous studies, researchers identified factors that affect women's autonomy in decision making regarding contraceptive choices at both the individual and community levels such as place of residence [20], age [15, 20–22], wealth index [15], women’s education [16, 22–24] and occupation [15, 16, 20, 24], religion [16], number of living children [20], desired to have children [25], media exposure [19], and visited health facility in the last 12 months [19].

A number of strategies, policies, and programs are in place at the global, regional, and national levels in order to improve safe motherhood. As a global movement toward universal health coverage, the SRH rights were recognized by the international conference for population development (ICPD) in 1994 and the millennium development goals (MDGs) in 2000 and the 2030 agenda for sustainable development goals (SDGs) [19].

In Ethiopia, laws and policies were revised in response to international conventions and human rights agreements reflected in ICPD, MDGs, and SDGs [19]. Implementing a 5-year Health Sector Transformation Plan strategy, as well as legalizing women's rights to information and protection from unwanted pregnancies, was some of the steps taken to improve reproductive health [19, 26, 27]. In spite of regulations and strategies designed to protect SRH and related rights, women still have a limited amount of autonomy over the use of contraceptives to prevent unintended pregnancies [28].

In high fertility regions, understanding the prevalence of decision making autonomy may assist in developing strategies aimed at empowering women's decision-making autonomy to use contraceptives. As these are regions of Ethiopia with higher fertility rate than WHO recommendations [29]. As a result, the current study concentrated on three high fertility regions in Ethiopia, as deferred to previous study that examined several other regions in combination [19]. Furthermore, in specific areas of high fertility regions of Ethiopia, women's autonomy in making decisions about contraceptive use have not yet been studied, which will aid policymakers and decision-makers in developing effective intervention. Therefore, the purpose of this study was to determine the prevalence of married women's decision-making autonomy on contraceptive use and the factors that influence in high fertility regions of Ethiopia.

## Methods

### Study settings and data source

**Fig. 1:**
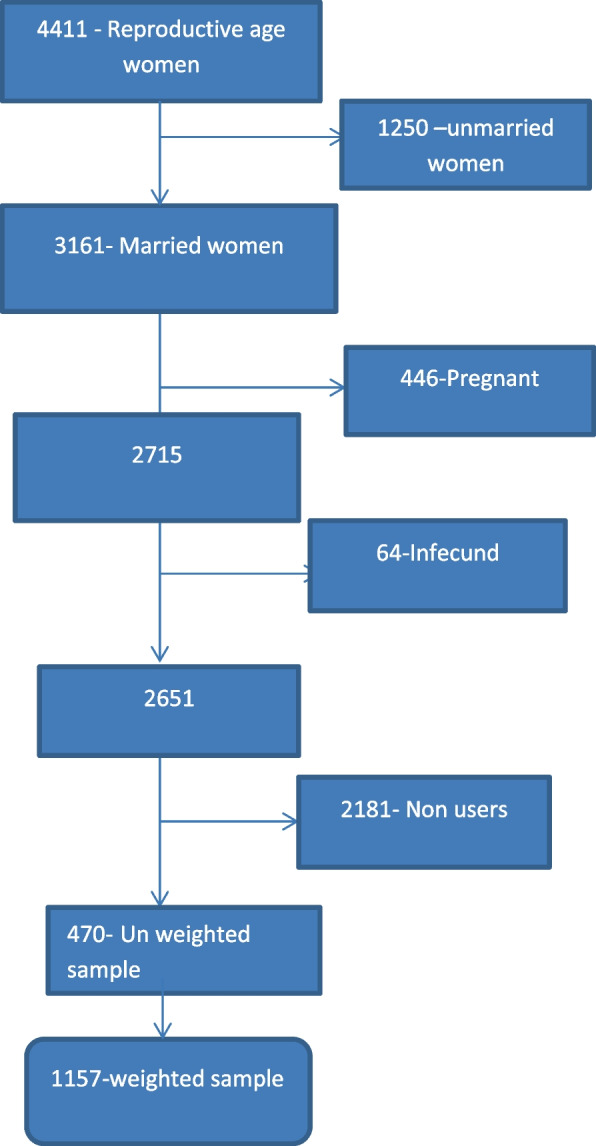
Schematic presentation of married women’s decision making autonomy on contraceptive utilization in high fertility regions of Ethiopia using EDHS data 2016

The study was a cross-sectional assessment of data from Ethiopian Demographic and Health Surveys (EDHSs) conducted from January 18, 2016, to June 27, 2016, across the country which is the fourth comprehensive survey [30]. Regions (Afar, Oromia, and Somali) were included in this study. These regions were selected because they are high fertility regions in Ethiopia with fertility rates above 5.0 a higher value than the rate of 4.6 in Ethiopia [29], 4.44 in SSA and 2.47 worldwide [31]. The data for these regions were gained from the official database of the EDHS program, www.measuredhs.com after authorization was granted via online request by explaining the purpose of our study. We extracted dependent and independent variables from the woman record (IR file). DHS is a nationally representative household survey conducted by face-to-face interviews on a wide range of populations. Study participants were selected using a two-stage stratified sampling technique. Enumeration Areas (EAs) were randomly selected in the first stage, while households were selected in the second stage [32]. For the sample data to be representative, weighting was conducted before analysis of the DHS dataset since households are not selected uniformly. We used the individual weight for women (v005), which is the household weight (hv005), multiplied by the inverse of the individual response rate. Individual sample weights were generated by dividing (v005) by 1,000,000 before analysis to approximate the number of cases [33, 34]. After exclusion of pregnant mothers, in fecund and who did not use contraceptives during survey, a total weighted sample of 1157 reproductive- age women was included from three regions in this study (Fig. 1).

### Study variables

#### Outcome variable

According to the study, "married women's independent decisions about contraception were the outcome variable." It was dichotomized into “not autonomous (No = 0)” (for married reproductive age women who reported that the decision on their contraceptive use was made mainly by her husband/partner, respondent and her husband/partner, and others) and “autonomous (yes = 1)” (for married reproductive age women who reported that the decision on their contraceptive use was made only by themselves) [5, 23].

### Independent variables

#### Individual level variables

Age of respondents, educational status of respondents, husband education, occupation of respondents, husband occupation, wealth index, media exposure, number of living children, age at first marriage,family/household size, and religion were included.

#### Community level variables

Community level variables included residence, region and some were derived from the individual level data of all community members in the primary sampling unit (PSU), which includes the community level poverty, community level education, and community level media exposure. Due to the non-normal distribution, we used the national median value to categorize these factors into high and low at community level. Education at the community level was determined by the proportion of households in the highest educated categories. Women are classified as low if less than 50% are educated, and as high if more than 50% are educated [35]. Poverty at the community level is calculated based on the proportion of households in the poorer and poorest quintiles. The proportion from a given community is grouped as low if it is less than 50% and as high if it is more than 50% [35].

#### Wealth status

(The variable wealth index was re-categorized as “Poor”, “Middle”, and “Rich” categories by merging poorest with poorer and richest with richer) [36–38].

#### Media exposure

(Media exposure was calculated by aggregating TV watching, radio listening, and reading newspapers and woman who has exposure to either of the media sources was categorized as having media exposure and the rest considered as having no media exposure) [39].

#### Multi-collinearity

In order to determine whether multi-collinearity exists among independent variables, the Variance Inflation Factor (VIF) was used using a cut-off value of 10. As a result, variables whose VIF value was less than 10 are considered to be non-multicollinear.

### Data analysis

For data analysis Stata version 16 software was used. The study tested with four models: the null model with no explanatory variables, model I with individual factors, model II with community factors, and model III with both individual and community factors. To compare and assess the fitness of nested models, we used the intra class correlation coefficient (ICC), the median odds ratio (MOR), and deviation (-2LLR). Model III was the best-fitting model due to its low deviance. In multivariable analysis, variables with a *p*-value less than 0.2 in bivariable analysis were used. Finally, in the multivariable analysis, adjusted odds ratios with 95% confidence intervals and *p*-values less than 0.05 were used to identify factors of decision making autonomy on contraceptive utilization.

## Results

### Individual level factors

More than half (54.4%) of the women were aged between 25–34 years. Regarding their educational level, 630 (54.4%) respondents were reported with no formal education. Among the participants, 790 (68.2%) had 1–4 living children. About 60.4% of the respondents were married at the age of 18 years above, 703 (60.8%) of participants has five to eight family size. With regard to their economic status, 285 (24.7%) women were from the poor wealth quintiles and 645 (55.7%) were from the rich wealth quintiles. Moreover, 477 (41.2%) respondents had media exposure. In addition 423 (36.6%) participants were Orthodox Christian (Table 1).

**Table 1:** Individual characteristics of respondents in high fertility regions of Ethiopia (*n* = 1157)

Variables	Categories	Frequency	Percentage (%)
Age of respondents	15–24	258	22.3
	25–34	629	54.4
	35–49	270	23.3
Educational status of respondents	No formal education	630	54.4
	Primary education	458	39.6
	Secondary and above	69	6.0
Husband education	No formal education	428	37.0
	Primary education	492	42.5
	Secondary and higher	237	20.5
Occupation of respondents	Working	480	41.5
	Not working	677	58.5
Husband occupation	Working	116	10.0
	Not working	1041	90.0
Wealth index	Poor	285	24.7
	Middle	227	19. 6
	Rich	645	55.7
Media exposure	Yes	477	41.2
	No	680	58.8
Number of alive children	No child	64	5.5
	1–4	790	68.2
	5–8	290	25.1
	≥ 9	13	1.2
Age at first marriage	< 18 years	698	60.4
	≥ 18 years	459	39.6
Family/Household size	1–4	390	33.7
	5–8	703	60.8
	9 and above	64	5.5
Religion	Orthodox Christian	423	36.6
	Muslim	409	35.4
	Protestant	300	25.9
	Others +	24.4	2.1

### Community level factors

Of the study participants, 922 (79.7%) were resides in rural area. Majority of participants (94.6%) of the reproductive age women were from communities with low proportion of community level poverty. Majority (80.9%) of participants had community media exposure. Moreover, 1141(98.6%) participants were from Oromia region (Table 2).

**Table 2:** Community level characteristics of respondents in high fertility regions of Ethiopia (*n* = 1157)

Variables	Categories	Frequency	Percentage (%)
Residence	Urban	235	20.4
	Rural	922	79.7
Community-level poverty	Low	1095	94.6
	High	62	5.4
Community media exposure	Low	221	19.1
	High	936	80.9
Community level education	Low	295	25.6
	High	861	74.4
Region	Afar	172	14.89
	Oromia	943	81.48
	Somali	42	3.63

### Prevalence of married women’s decision making autonomy on contraceptive use in high fertility regions of Ethiopia

**Fig. 2:**
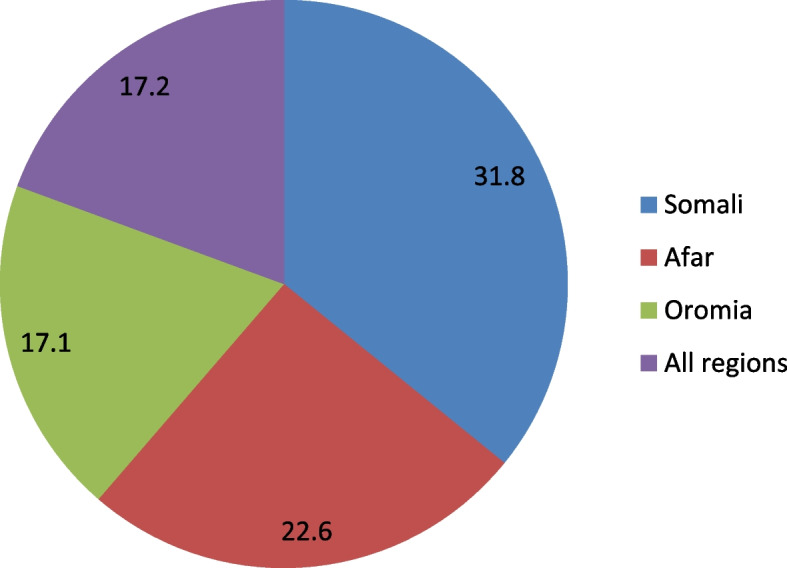
Prevalence of married women’s decision making autonomy on contraceptive use in high fertility regions of Ethiopia, 2016 EDHS data

Overall, the prevalence of married women’s decision making autonomy on contraceptive use in high fertility regions of Ethiopia was 17.2% (95% CI: 15.1, 19.5) (Fig. 2).

### Random effects and model fitness

According to the intra-class correlation (ICC) in the null model, 59% of the overall variability of decision making autonomy can be attributed to cluster variability. The median odds ratio for decision making autonomy in the null model was 5.9, indicating that decision making autonomy varied between clusters. This suggests that if we randomly picked individuals from different clusters, those in the highest decision making autonomy cluster had a 5.9 times higher chance of possessing decision making autonomy than those in the lowest decision making autonomy cluster. Likewise, the proportional change in variance (PCV) increased from 13.8% in model I to 61.4% in model III (a model with individual and community variables), which indicates that the final model (Model III) best describes decision making autonomy variability. Deviance was also used to assess the fitness of the model. Model III was found to have the lowest deviation, so it was the best fitting model (Table 3).

**Table 3:** Model comparison and random effect analysis result in high fertility regions of Ethiopia

Random effect	Null Model	Model I	Model II	Model III
Variance	5.23	4.51	2.58	2.02
ICC	59.04	50.76	43.16	31.7
MOR	5.9	5.6	4.2	3.1
PCV	Ref	13.8%	50.7%	61.4%
**Model fitness**				
Deviance (-2LLR)	956	842	908	812

### Factors associated with married women’s decision making autonomy on contraceptive use

In terms of individual level factors, the study showed that women aged 25 to 34 and 35 to 49 were more likely to have decision making autonomy on contraceptive (AOR = 3.19; 95% CI:1.55, 6.54), (AOR = 3.59; 95% CI: 1.5, 8.36) compared to 15 to 24 aged women respectively. The study found that women with a secondary and above educational level were 3.38 times more likely decision making autonomous on contraceptive use (AOR = 3.38; 95% CI: 1.07, 10.67) than those who had no formal education. Women who follow Muslim in religion were 53% less likely decision making autonomous on contraceptive use (AOR = 0.47; 95% CI: 0.23, 0.98) than Orthodox Christian followers. Women who had their first marriage before the age of 18 years were less likely to have decision-making autonomy on contraceptive use compared to women who married at 18 years or above (AOR = 0.42; 95% CI:0.26, 0.68).

With regard to the community level factors, the odds of decision-making autonomy on contraceptive use was high among women who had community media exposure (AOR = 1.85; 95% CI: 1.15, 2.48) compared with unexposed to media. Moreover, women who were residing in urban area were 2.73 times more likely decision making autonomous on contraceptive use than living in rural area (AOR = 2.73; 95% CI: 1.97, 6.35) (Table 4).

**Table 4:** multivariable analyses for factors affecting married women’s decision making autonomy on contraceptive use in high fertility regions of Ethiopia **(***n*** = **1157)

Variables	Model 0	Model 1 AOR (95% CI)	Model 2 AOR (95% CI)	Model 3 AOR (95% CI)
Individual level Characteristics				
Age				
15–24		1		1
25–34		4.21 (2.12, 8.38)		3.19 (1.55, 6.54)^a^
35–49		5.00 (2.21, 11.28)		3.59 (1.5, 8.36)^a^
Educational status of the respondents				
No formal education		1		1
Primary education		0.85 (0.50, 1.45)		1.06 (0.60, 1.85)
Secondary and above		1.81(0.66, 4.95)		3.38 (1.07, 10.67)^a^
Wealth index				
Poor		1		1
Middle		0.41(0.20, 0.81)		0.50 (0.25, 1.00)
Rich		0.52 (0.27, 0.976)		0.53 (0.26, 1.03)
Media exposure				
No		1		1
Yes		1.24 (0.73, 2.11)		1.30 (0.74, 2.28)
Age at first marriage				
≥ 18 years		1		1
< 18 years		0.37 (0.21, 0.53)		0.42 (0.26, 0.68)^a^
Number of alive children				
No child		1		1
1–4		2.01(0.65, 6.20)		2.77 (0.84, 9.19)
5–8		2.07 (0.56, 7.64)		3.37 (0.84, 13.6)
≥ 9		0.03(0.001, 1.543)		0.05 (0.36, 6.17)
Religion				
Orthodox Christian		1		1
Muslim		0.17 (0.08, 0 .37)		0.47 (0.23, 0.98)^a^
Protestant		0.54 (0.26, 1.10)		0.18 (0.08, 1.40)
Others +		0.01(0.006, 1.75)		0.01 (0.07, 2.03)
Community level variables				
Community level poverty				
High			1	1
Low			1.17 (0.18, 7.56)	1.07 (0.15, 7.69)
Community media exposure				
Low			1	1
High			0.59(0.15, 2.30)	1.85 (1.15, 2.48)^a^
Residency				
Rural			1	1
Urban			2.95 (0.78, 11.15)	2.73 (1.97, 6.35)^a^

Null model: adjusted for individual-level characteristics, Model 2: Adjusted for community-level characteristics, Model 3: adjusted for both individual and community level characteristics

Others + : catholic, traditional, and other EDHS category

^a^Statistically significant, AOR Adjusted Odds Ratio, COR Crude Odds Ratio

## Discussion

This study sought to ascertain married women's decision-making autonomy regarding contraceptive use and associated factors in high fertility regions of Ethiopia. The current study revealed that below one fifth (17.2% (95% CI: 15.1, 19.5)) of married women in high fertility regions of Ethiopia had contraceptive decision-making autonomy. This finding is lower than studies conducted in Northwest Ethiopia 77.3% [40], Adwa North, Ethiopia 35.9% [23], Ethiopia 21.6% [19], South Africa 22% [16], and Nigeria 24% [13]. This discrepancy could be explained by variations in the sample size, as well as socioeconomic and sociocultural aspects of society. Additionally, it can be due to variations in the study population and methodological approaches used by other studies.

The odds of decision-making autonomy on contraceptive use among women aged 25–34 and 35–49 years were more likely to have decision making autonomy than those women aged 15–24 years. This result is consistent with earlier research conducted in Ethiopia [5, 15, 20] in which older respondents reported more autonomy in decision-making on contraceptive use. This finding can be explained by the fact that younger women have less influence over their contraceptive decision because they are less likely to visit family planning clinics and have restricted access to SRH information [41].

According to this study, women with secondary-level or higher education were more likely to have decision making autonomy on use of contraceptives than those women without formal education. This finding is in line with other studies such as a study conducted at Mizan-Aman, Ethiopia [24] and Nigeria [13, 42]. As a possible justification, education empowers women to be independent and provides them with essential information that can be crucial in determining their reproductive health. In addition, educated women are more likely to make collective decisions with their husbands regarding their health, children's health, and visits to relatives or family members, which is beneficial to sharing experiences and exercising their reproductive and human rights.

Women who were Muslim were less likely to have decision-making autonomy on contraceptive use than Orthodox religious followers. This result is in line with the findings of a study conducted in Ethiopia [19], South Africa [16] and Ghana [43] which discovered that fellows of the Christian religion had better odds of making their own decisions about using contraceptive. There might be a reason for this as the Islam religion allows polygamy, and most of the women who follow this faith believe they can gain a husband's attention by getting pregnant for him [44]. The likelihood of using modern contraceptives decreases when you are in a polygamous relationship [45].

Women who had their first marriage before the age of 18 years were less likely to have decision-making autonomy on contraceptive use compared to those married at 18 years or above. It is consistent with a previous study [19], that found early marriage decreases women's decision making autonomy when it comes to contraceptive use. Possibly, this is because young women have less negotiating power and limited educational opportunities due to early marriages [19, 46].

Women with high community media exposure to family planning messages were more likely to have decision-making autonomy on contraceptive use compared to women with low community media exposure to family planning messages. This finding is consistent a study conducted in Ethiopia [19]. This finding may be explained by the fact that women who are exposed to family planning messages in the community media frequently may have a better understanding of their rights regarding reproductive health and the benefits of using health care services, which promotes their involvement in reproductive health decisions [19].

Moreover, urban women have greater liberty in making decisions about using contraception than rural women. This is in line with studies conducted in Ethiopia [19, 20]. The possible justification might be that urban women are more educated and have better community exposure to family planning messages than their counterparts. In addition, urbanization allows them to have greater involvement in the household decision-making process and to decide on contraceptive use [47, 48].

To account for the data's hierarchical structure, we employed a mixed-effect model, multilevel analysis (advanced model). However, due to the cross-sectional character of the study, it is impossible to draw cause-and-effect linkages between the dependent variable and the independent variables. EDHSs are based on self-reported data, which because of its socio-cultural context is probably subject to social desirability bias. The fact that the current study employed data acquired from the EDHS precludes triangulation of data using various data gathering methods, which is another weakness of the study. In addition as we used secondary data, only a few samples met the different exclusion criteria, so generalizing might be challenging.

## Conclusion

According to this study, married women’s decision-making autonomy on contraceptive use was low. Age of respondents, educational status of mothers, age at first marriage, place of residence, religion, and community media exposure were significant factors for decision making autonomy. Therefore, the government should promote women’s autonomy on contraceptive use as an essential component of reproductive health rights through mass media, educating, with particular attention for, youth women, and women living in rural settings.

## Data Availability

Data for this study were sourced from Ethiopian Demographic and Health surveys (EDHS), which is freely available online at (https://dhsprogram.com).
